# (Not) part of the team: Racial empathy bias in a South African minimal group study

**DOI:** 10.1371/journal.pone.0283902

**Published:** 2023-04-06

**Authors:** Melanie Deist, Melike M. Fourie

**Affiliations:** 1 Centre for the Study of the Afterlife of Violence and the Reparative Quest, Stellenbosch University, Stellenbosch, South Africa; 2 Neuroscience Institute, University of Cape Town, Cape Town, South Africa; University of Salford School of Health and Society, UNITED KINGDOM

## Abstract

Minimal Group Paradigm (MGP) research suggests that recategorization with an arbitrarily defined group may be sufficient to override empathy biases among salient social categories like race. However, most studies utilizing MGPs do not consider sufficiently the socio-historical contexts of social groups. Here we investigated whether the recategorization of White participants into arbitrarily defined mixed-race teams using a non-competitive MGP would ameliorate racial empathy biases towards ingroup team members in the South African context. Sixty participants rated their empathic and counter-empathic (*Schadenfreude*, *Glückschmerz*) responses to ingroup and outgroup team members in physically painful, emotionally distressing, and positive situations. As anticipated, results indicated significant ingroup team biases in empathic and counter-empathic responses. However, mixed-race minimal teams were unable to override ingroup racial empathy biases, which persisted across events. Interestingly, a manipulation highlighting purported political ideological differences between White and Black African team members did not exacerbate racial empathy bias, suggesting that such perceptions were already salient. Across conditions, an internal motivation to respond without prejudice was most strongly associated with empathy for Black African target individuals, regardless of their team status. Together, these results suggest that racial identity continues to provide a salient motivational guide in addition to more arbitrary group memberships, even at an explicit level, for empathic responding in contexts characterized by historical power asymmetry. These data further problematize the continued official use of race-based categories in such contexts.

## Introduction

An enduring legacy of apartheid South Africa is the artificial racial categories that were engineered with the objective to benefit White people while discriminating against Black people (including Black African, Coloured, and Indian people) (Population Registration Act, 1950). While the power dynamics have shifted since 1994, with a ruling party that is now representative of the Black majority, these socio-historical race categories remain in use as part of government policy to address historically unjust racial inequalities [1]. Yet measures of redress have not rebalanced the scales, as the White minority continue to hold significant economic power and privilege, while wealth and income inequalities continue to be rampant within Black communities [2–4].

In some ways, apartheid South Africa could be viewed as a large, albeit ethically deeply inhumane, social laboratory—forging racial categories that maximized differences and response biases between groups that continue to be resistant to change today [5]. Racial categorization is socially constructed, however, and serves political goals, rather than carrying inherent or biological meaning [6]. As with other social categories, racial categories are therefore always in flux and amenable to manipulation. Indeed, abundant research in social psychology suggests that recategorization with an arbitrarily defined group may be sufficient to override racial response biases across perceptual, emotional, cognitive, and behavioral domains [7–9]. The question we grapple with here, however, is whether recategorization via the Minimal Group Paradigm is sufficient to negate deeply-rooted response biases inherent to contexts with protracted conflict and historical social hierarchies?

### Empathy and the Minimal Group Paradigm

The Minimal Group Paradigm (MGP) is arguably one of the most influential strategies to impact intergroup relations in the absence of realistic conflicts of interest [10]. It involves assigning participants to novel groups based on some arbitrary criteria (e.g., whether they over or underestimate the number of dots on a screen), after which participants then typically report heightened ingroup identification and respond with ingroup biases on a variety of tasks. The success of the strategy could be attributed to the fact that social group biases (including racial biases), appear to depend heavily on the perceiver’s social motivation, which is context sensitive and can be modulated [11, 12] (for a review, see [13]). Membership in a mixed-race team thus appears to align people’s motivation in favor of the ingroup, regardless of its (racial) make-up. Some evidence suggests that the MGP is effective also in modulating implicit or neural responses, overriding visually salient race cues in favor of the ingroup team [14–17] (however, see also [18]).

A salient finding in intergroup contexts is that people respond with greater empathy when they share a social category with the person in distress, and stifled empathy when the person does not belong to their ingroup [19]. This phenomenon has variously been described as “an intergroup empathy gap”, “intergroup empathy bias”, or “parochial empathy” [20–22], and consistently shapes both behavioral and neural indices of empathic responding [23–25]. Impaired empathy for a social outgroup may mediate a host of negative outcomes, including reduced prosocial action and passive harm, which exacerbates intergroup conflict [26, 27]. Ameliorating intergroup empathy bias is therefore of great import.

Arbitrary group categorization by way of the MGP also appears effective in modulating empathic responses [28]. For example, the mere assignment of participants into non-relevant social groups appears sufficient to facilitate an ingroup bias in empathy for physical pain [29]. Similarly, across a series of studies, Cikara and colleagues [30] demonstrated that pitting novel teams against each other not only decreased empathy for the outgroup relative to the ingroup team, but also increased counter-empathic responses. Hence, compared to ingroup team members, participants responded with more pleasure when outgroup team members experienced adversity (*Schadenfreude*), and more displeasure when outgroup team members experienced success (*Glückschmerz*). Moreover, a recent study showed that ingroup activation biases in empathy-related brain regions associated with established social categories (e.g., religion) could be extended also to categories formed more arbitrarily (e.g., novel religious teams at war) [31], providing evidence that neural empathic responses can be modulated flexibly by group affiliation.

Not many studies employing the MGP in empathic responding have included race as a variable, however, and results from those that have are inconsistent. In two studies examining Chinese students’ neural empathic responses to physical pain expressed by Chinese and Caucasian faces, for example, the temporary group relationship formed through mixed-race teams seemed sufficient in reducing racial bias in neural empathic response to ingroup team members [32, 33]. Other studies, however, showed that racial biases in neural empathic responses were unaffected by minimal forms of group categorization [34, 35]. Specifically, whereas participants showed clear team biases (unaffected by race) on explicit group identification and implicit affective priming tasks, neural empathic response was indicative of an own-race bias [35]. A working hypothesis for this broader area of study might therefore be that the MGP is more effective in overriding racial response biases on measures over which one has some degree of conscious control (i.e., behavioral, implicit) than those measures beyond the scope of conscious control (i.e., neural activation).

### Contextual factors

#### Historical power dynamics

In contexts where race plays a significant role in group distinction, racial identity—and the multiple features of intergroup relations that covary with racial identity, such as majority/minority status—may provide an equally salient motivational guide for perception and behavior (including empathic responses) as group membership [36, 37]. Yet, despite the efficacy of MGPs in modulating racial biases under certain conditions, most studies utilizing the MGP do not sufficiently take into account the social contexts in which social groups exist [32–36]. Historically-embedded social hierarchies, for example, may continue to impact current intergroup relations and interfere with MGP categorization [37, 38].

Racialized hierarchies tend to shape social identities, which, in turn, influence attitudes and behaviors towards racial outgroups [36, 39]. Because groups at the top of hierarchical social structures often enjoy better educational, financial, occupational, and health outcomes, they have a direct stake in maintaining the hierarchical status quo [40–42]. Lower-status groups may therefore be perceived as potential threats to societal structures that safeguard ingroup privilege, which could bring forth status keeping behavior that reinforce hierarchical group boundaries [36, 39, 43].

Given the above, it is unclear whether arbitrarily-defined mixed-race teams (as per the MGP) are a reliable means to override racial empathy biases, even at an explicit level, in social contexts with an extended history and continuing legacies of asymmetric structural relations. In South Africa, the role of race in historical social hierarchies and contentious power dynamics may contribute to shifting group categorization from team membership back to race, thus eliciting racial empathy bias.

#### Racialized political ideologies

Another contextual factor that could limit the efficacy of recategorization via the MGP, is the public salience of racialized political ideologies. Previous work has demonstrated the influence of ideological salience on intergroup relations and the importance of understanding cognitive processes within larger ideological contexts [44–46]. Opposing political ideologies have also been shown to modulate empathic responses, with greater empathy reported between individuals with similar political ideologies [47, 48].

In the South African context, political narratives are often racialized and shared in ways that arouse, rather than inform, their audience [49, 50], potentially increasing intergroup threat [36, 51, 52] and reinforcing racialized group boundaries. Two salient examples include the sensationalistic manner in which mainstream and social media regularly report on heated political debates surrounding Broad-based Black Economic Empowerment (BBBEE) and land expropriation without compensation. Whereas the BBBEE Act of 2013 aims to redress the apartheid legacies of Black economic exclusion [53, 54], the land redistribution program centers around the equitable transfer of underutilized, White-owned land to previously disadvantaged population groups in response to the historic forced removals of Black South Africans from their land [55]. Both political policies thus aim to redress past imbalances, yet are often depicted in inflammatory ways that incite racial polarization and intergroup threat (e.g., “If you see a beautiful piece of land, take it–Malema”, News24, 28-02-2017, [56], see also [57, 58]). Moreover, political parties tend to weaponize these policies to sow racial division and gain political traction [49, 50].

It may thus be reasonable to predict that when such racialized political ideologies are afforded greater saliency, it will contribute to greater racial empathy bias, also under MGP conditions.

#### Individual differences

Beyond the social structures that might influence empathic responding, individual beliefs and attitudes can also influence racial empathy bias [59]. Notably, converging evidence suggests that racial response bias could be influenced by personal motivations to appear non‐prejudiced [60, 61]. In the South African context, White participants motivated by external societal pressures to respond without prejudice were associated with reduced neural activation in key empathy areas in response to racial outgroup members [62], whereas in another study, participants with greater internal motivation to appear non‐prejudiced responded with greater prosocial helping behavior toward racial outgroup members [63].

Acknowledging the extent of historical suffering experienced by the outgroup may also attenuate cross-racial empathic response bias [64]. Hughes and colleagues [65], for example, found that European American children expressed less racial bias after learning about historical racism experienced by African Americans. Indeed, various studies show that acknowledging the impact of historical suffering on continuing racialized inequality fosters support for social change and transformational redress [25, 66, 67].

### Study aims

Here we investigated whether findings from empathy research using the MGP replicate within the South African context, given its oppressive socio-political history and continuing asymmetric structural relations. Specifically, we adopted a non-competitive MGP to examine whether the recategorization of historically advantaged White individuals (perceived racial ingroup, and racial group with whom the authors identify—see also *Positionality Statement*) into artificial mixed-race teams with Black African individuals (perceived racial outgroup) would be effective in overriding potential ingroup racialized response biases in empathy for physically painful, emotionally distressing, and positive events [68, 69]. We distinguished between empathy for these event types because previous research suggests they may be separable capacities with distinct affective, behavioral, and physiological markers [70–73]. In addition, we examined whether cross-racial empathic responding within arbitrarily-defined teams is affected when racial groups’ political ideological differences are made salient, as is often done in the media.

The participant group in the present study consisted of second-generation White students enrolled at a historically White university. Despite significantly greater diversification in the demographic composition of the student body at such higher education institutions post-apartheid, they often remain highly racialized spaces in which power inequalities, exclusionary practices, and racism persist [74–76]. Exploring the flexibility of group categorization and resulting empathic responding in such settings is therefore of great interest. In using racial categories, our intention is not to essentialize or reify race [77], but rather to unmask the influence of apartheid on specific groups of people and to accentuate the continued social implications of racial identity [78, 79]. In this spirit, race labels are employed to refer to groups of people whose lives have been substantially marked by the meanings of these labels, in full recognition of the complexities and heterogeneities that they represent [80].

We constrain our use of the word empathy to refer to an other-oriented affective reaction elicited by and congruent with the perceived well-being of someone experiencing either a negative or positive event, and counter-empathic responses as being incongruent with that person’s welfare [81]. Based on previous research, we hypothesized that (i) White participants will exhibit an ingroup team bias, such that empathy ratings will be greater (and counter-empathy ratings will be reduced) for ingroup compared to outgroup team members. We further anticipated that (ii) the MGP will reduce (but not override) racial empathy bias for ingroup team members, but not for outgroup team members, and that (iii) a racial political salience manipulation, where politically divisive ideologies are made salient, will have the effect of reinstating racial empathy bias amongst ingroup team members.

We also examined associations between participants’ individual difference characteristics and their degree of empathic responding towards racial outgroup members, regardless of team status. Here we hypothesized that (iv) a higher internal motivation (IMS) to respond without prejudice will be associated with greater empathy for racial outgroup members, whereas a higher external motivation (EMS) to respond without prejudice will be associated with reduced empathy for racial outgroup members. Finally, we predicted that (v) greater perceptions of Black historical suffering will be associated with greater empathy for racial outgroup members.

## Materials and procedures

### Participants

A power analysis using G*Power [82] indicated that a sample size of 58 participants would be sufficient to detect a moderate to low effect size (0.3) with reasonably good power (0.8) at an α level of .05. Sixty self-identified White, undergraduate students (50% female, *M*_*age*_ = 20.35 years, *SD*_*age*_ = 1.62 years) participated in the present study and received ZAR50 (~ 4 USD) as compensation for their time (see S1 Text for further sampling information). Participants were randomly assigned to the control (*n* = 30) or manipulation (*n* = 30) conditions. All study procedures were approved by the University of Stellenbosch’s Humanities Research Ethics Committee and the research was carried out according to these guidelines.

### Experimental design

**Fig 1:**
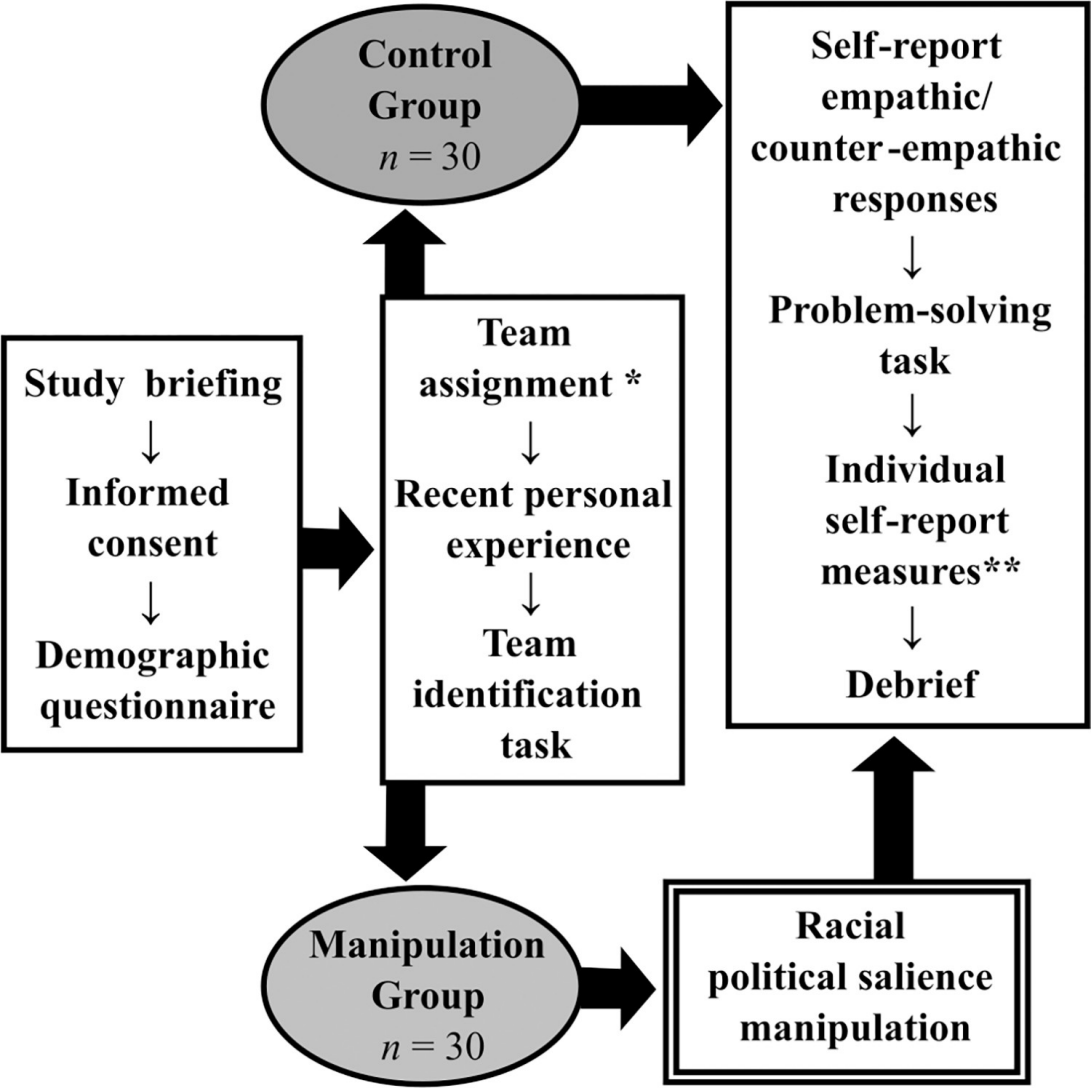
Flow diagram detailing the data collection procedure. *All participants were assigned to the Eagles team. ** These included the (i) Internal and External Motivation to Respond Without Prejudice scales (IMS/EMS) [83]), and (ii) perceptions of Black African and White historical suffering.

We employed a 2 (racial political salience: control vs manipulation) x 2 (target team: Eagles vs Leopards) x 2 (target race: Black African vs. White) repeated measures multi-factorial design. The MGP was both blinded and non-competitive, and adapted from Cikara and colleagues [30]. A flow diagram of the data collection procedure is illustrated in Fig 1.

First, to examine whether the MGP would override racial empathy bias for ingroup team members, participants were ostensibly assigned to one of two mixed-race teams, Eagles or Leopards. In reality, all participants were assigned to the Eagles team. They then completed a task measuring their empathic and counter-empathic reactions to members of both teams in physically painful, emotionally distressing, and positive situations.

Second, half of participants were also assigned to a racial political salience manipulation. Participants in this condition were asked to rate their support for five online petitions, some of which were linked to highly racialized political policies. They were then presented with diagrams supposedly representing the polarized petition support of White and Black African members of the Eagles and Leopards teams. These diagrams were fictitious, however, and did not reflect the opinions (or images) of any study participants. Rather, the diagrams were designed to highlight purported political ideological differences between White and Black African team members, making these identities more salient. We hypothesized that spotlighting these differences would exacerbate racial empathy bias amongst ingroup team members.

#### Team assignment

A dot-estimation task (adapted from Montalan et al. [29]) was used ostensibly to assign participants into two teams (i.e., Eagles or Leopards). Participants were shown a random display of dots for five seconds and were asked to write down the estimated number of dots (see stimulus in S1 Fig). Each participant was then assigned a cognitive profile supposedly based on whether they overestimated or underestimated the number of dots. In actual fact, all participants were assigned to the Eagles team. The results of the dot-estimation task were not recorded, given that the task simply served to give rise to a minimal group effect.

#### Team identification

Participants rated their identification with the teams by responding to two statements regarding each team (“I identify with the [Eagles/Leopards]” and “I feel connected to the [Eagles/Leopards]”) on a scale ranging from 1 (*strongly disagree*) to 9 (*strongly agree*). These items were highly correlated for each team (*r*s > .50, *p*s < .001), and thus averaged to create a composite identification score for the ingroup (Eagles) and outgroup (Leopards) team.

#### Racial political salience manipulation

In this manipulation we increased the salience of potential political ideological differences between White and Black African people to examine its effect on empathic responding, particularly with respect to the newly-formed racially-mixed ingroup (i.e., Eagles team).

Participants in the manipulation condition were presented with five supposedly real online petitions: one petition sought support against a progressive policy that would benefit historically disadvantaged racial groups (White-biased petition; “Urge parliament to tone down the Broad-based Black Economic Empowerment (BBBEE) Act, so that qualified White people stand a better chance of employment”); two petitions sought support towards more aggressive policies that would benefit historically disadvantaged racial groups at the expense of White people (Black-biased petitions; “Urge Stellenbosch University to expand free tertiary education for Black students at the expense of White students” and “Urge parliament to make urgent provision for the expropriation without compensation of White-owned land”), and two petitions sought support towards more general, politically neutral, campus issues, namely water saving efforts and drinking and driving (arbitrary petitions—see stimulus in S1 Text). These petitions were presented to participants using E-Prime 3.0 software [84] and participants were asked to rate their support for each petition using a visual analogue scale (VAS) ranging from 1 (*not at all*) to 7 (*very much*).

**Fig 2:**
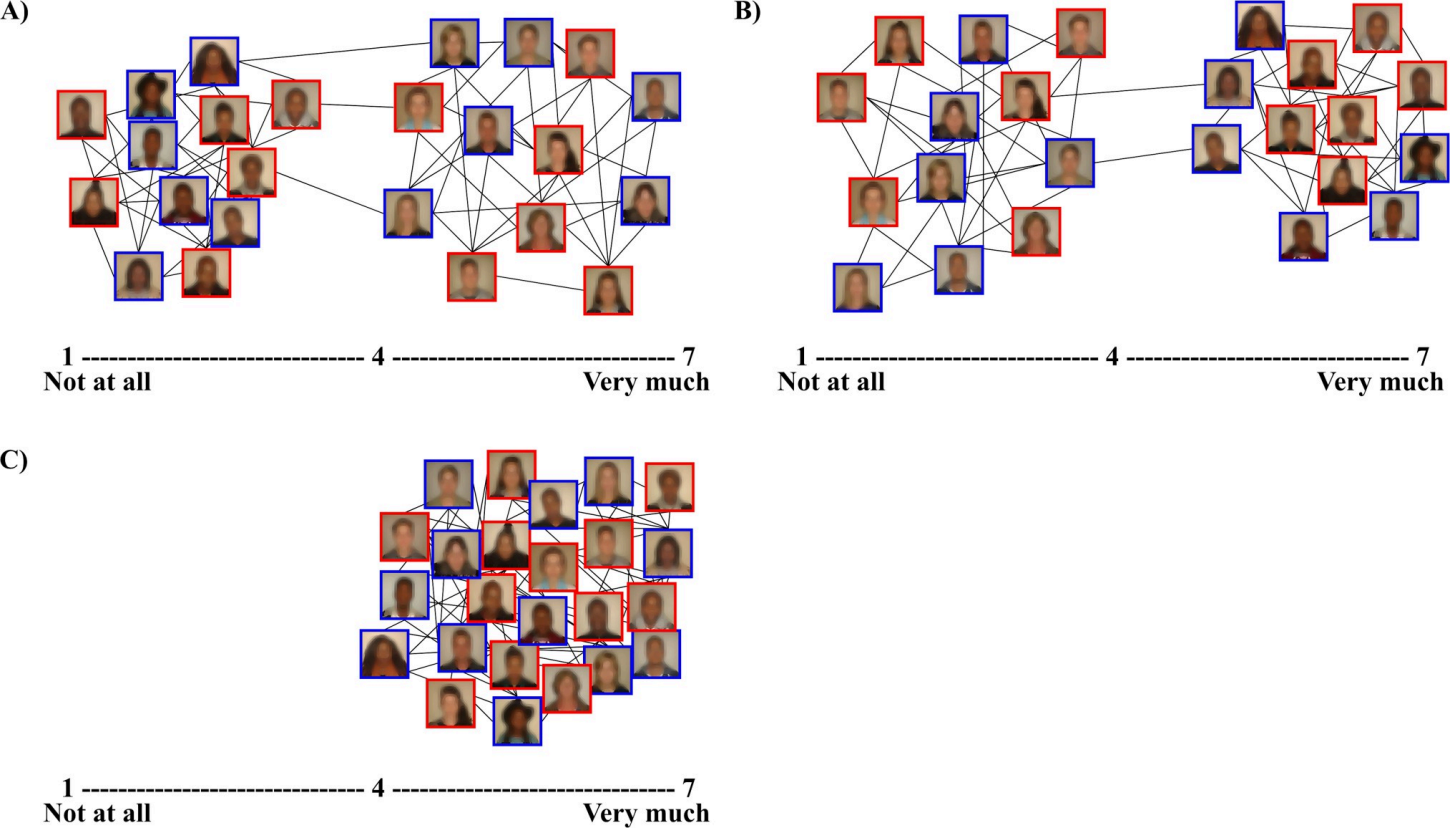
Sample diagrams of the racial political salience manipulation. A) The diagram indicated that White team members were largely in support of a petition that urged parliament to revoke a progressive policy that would benefit historically disadvantaged groups. B) The diagram indicated that Black African team members were largely in support of petitions that urged parliament to implement aggressive policies at the expensive of previously advantaged White people. C) The diagram indicated broad support from all team members towards arbitrary petitions about general campus matters. *Note*. None of the individuals in these images participated in the current study.

Once they completed each rating, participants were shown a diagram supposedly representing petition support amongst other Eagle and Leopard team members (see Fig 2A–2C). The diagrams indicated that White individuals were largely in support of “White-biased petitions” and Black African individuals were largely in support of “Black-biased petitions”, irrespective of their team membership, whereas all individuals supported the arbitrary petitions.

#### Empathy task

Empathy and counter-empathic responses were assessed using a self-reported reaction task that measured how good/bad participants felt about the fortunes/misfortunes of others. Each participant was presented with 24 events (8 physically painful events, e.g., “James’s fingers got slammed in a car door”/“Anele cut her foot on broken glass”; 8 emotionally distressing events, e.g., “Nicola and her best friend haven’t spoken in months”/“Thandi hasn’t seen her mother in a year”; and 8 positive events, e.g., “Siyanda scored a goal for his soccer team”/“Nico found a R50 note on the floor”) using E-Prime 3.0 software [84] (for a full list of stimuli, see S1 Text).

Each event type was randomly paired with the photograph of the target individual who supposedly experienced the event. These photographs were not photos of actual participants in the study, but were collected with prior consent from random students on campus and included 12 Black African and 12 White individuals (six males and six females in each racial group). To make the group identity salient, each photograph was accompanied by a background color (red or blue) and team logo (Eagle or Leopard) signifying supposed team membership. The team membership, event type, and team logo color were counterbalanced for each target individual across participants and the presentation order was randomized.

After each event, participants were asked to indicate: (1) “How sorry does this make you feel?” and (2) “How good does this make you feel?” on continuous VAS sliders ranging from 1 (*not at all*) to 9 (*extremely*). Empathic responses for physically painful and emotionally distressing events were assessed with “sorry” ratings, whereas counter-empathic responses were assessed with “good” ratings. For positive events, empathic responses were assessed with “good” ratings, whereas counter-empathic responses were assessed with “sorry” ratings.

#### Motivation to respond without prejudice

The Internal Motivation Scale (IMS) and External Motivation Scale (EMS) [83] were used to determine internal (e.g., “I attempt to act in nonprejudiced ways toward Black African people because it is personally important to me”) and external motivations (e.g., “Because of today’s PC (politically correct) standards I try to appear nonprejudiced toward Black African people”) to respond without prejudice. Each item was measured on a Likert-type scale, ranging from 1 (*strongly disagree*) to 9 (*strongly agree*). Higher IMS scores indicate a higher likelihood that a participant’s reason for responding without prejudice is due to personal motivations, whereas higher EMS scores indicate a higher likelihood that a participant’s reason for responding without prejudice is due to external social motivations.

#### Perceptions of historical suffering

To assess perceptions of historical suffering, participants were asked to respond to the questions “Historically, how much suffering has Black African people in South Africa endured?” and “Historically, how much suffering has White people in South Africa endured?” These questions were rated on Likert-type scales ranging from 1 (*not much at all*) to 9 (*very much*). Higher scores thus indicated greater perceptions of historical suffering.

### Procedure

During recruitment, the study was presented as a problem-solving challenge. Similar to the cover story used by Cikara and colleagues [30], prospective participants were told that the study purpose was to examine whether knowledge about team members enhances team performance in subsequent problem-solving tasks. Participants were thus blinded to the true focus of the present investigation to minimize socially desirable responses.

All procedures were conducted in private computer rooms on campus. Upon arrival, experimental procedures were explained and written informed consent was obtained from all participants.

Participants were first asked to complete a short demographic questionnaire reporting their age, gender, racial group they identify with (inclusion criteria), and home language.

Next, they were told the following cover story: “*The goal of our study is to assess cognitive functioning in teams and whether knowledge about team members enhances team performance in subsequent problem-solving challenges*. *We will be assigning you to a team (either Eagles or Leopards) with a similar cognitive processing profile in a moment*. *Each week*, *for the duration of the data collection period*, *these two teams participate in a series of cognitive problem-solving challenges*. *Though they are not in competition with each other (thus independent)*, *we want to see whether knowing something personal about your fellow team members will enhance your performance*. *So*, *some participants will read about random news events*, *and some will read about team members’ recent experiences*. *You are in the latter category*. *When a team successfully completes 50 tasks before the end of the semester*, *all members of that team will win extra cash*. *You will be contacted in due course if this happens*.*”*

After this briefing, participants completed the dot-estimation task to supposedly divide them into two teams (Eagles or Leopards) based on their cognitive profile (see S1 Fig). They were either identified as *under-estimators (*individuals who supposedly underestimated the number of dots in the dot-display) or *over-estimators* (individuals who supposedly overestimated the number of dots in the dot-display). Participants were assured that neither of the two cognitive profiles were deemed better than the other and that it simply influenced how they processed information. Unbeknownst to them, however, all participants were assigned to the Eagles team.

After team assignment, participants completed the computerized team identification task. Furthermore, in line with the cover story, participants were asked to write down a recent experience (either positive or negative) that would supposedly be read by future team members.

This was followed by the manipulation task for the 30 participants randomly assigned to this condition, with the supporting cover story: *“As part of getting to know the Eagles and Leopards team members*, *you will be presented with a list of petitions on recent social issues that investigate social attitudes*. *You will be asked to indicate the extent to which you support each statement from 1 (not at all) to 7 (very much)*. *After indicating the extent to which you support each petition*, *you will be shown a network diagram of the extent to which other Eagles and Leopards team members have supported each petition*.*”* The three petitions of interest (i.e., 1 White-biased petition and 2 Black-biased petitions) were followed by diagrams supposedly indicating the racially polarized manner in which other Eagles and Leopards team members had responded to the petitions, whereas the arbitrary petitions were followed by diagrams indicating broad support from all team members (see Fig 2). Those participants in the control condition neither participated nor received any feedback on this task.

Next, participants across both conditions were presented with the computerized empathy task and asked to rate the extent to which they felt “sorry” and/or “good” about recent physically painful, emotionally distressing, and positive experiences of other ingroup (Eagles) and outgroup (Leopards) team members. Each participant was randomly presented with 24 scenarios—8 representing physically painful events (4 ingroup team members: 2 Black African, 2 White, and 4 outgroup team members: 2 Black African, 2 White), 8 representing emotionally distressing events, and 8 representing positive experiences.

Following these measures, participants were presented with timed problem-solving tasks not analyzed here, which supported the cover story (see S1 Text). We emphasized that the problem-solving tasks was not a competition between teams and that each team that successfully completed 50 tasks in the assigned time frame will win extra cash. Finally, participants completed measures assessing motivation to respond without prejudice and perceptions of historical suffering.

All participants were probed for suspicion before being debriefed fully. They were granted the opportunity to ask questions and allowed to withdraw their data should they wish to (none did). Participants were asked not to discuss the study and data collection methods with other students.

## Results

### Manipulation checks

#### Team manipulation

The efficacy of the team manipulation was assessed by analyzing the extent to which participants identified with the ingroup (Eagles) versus the outgroup (Leopards) team. A paired-samples *t*-test indicated that participants’ ingroup team identification score (*M* = 7.16, *SD* = 0.73) was significantly greater than their outgroup team identification score (*M* = 2.15, *SD* = 0.43), *t*(59) = 40.54, *p* < .001, *d* = 5.23. The team manipulation thus appeared successful.

#### Racial political salience manipulation

In the manipulation condition, participants responded to the five petitions as expected: the petition that would benefit White people, yet negatively impact historically disadvantaged people (i.e., White-biased petition) was strongly supported, whereas those petitions that would advance equality (i.e., Black-biased petitions) went unsupported. Specifically, support for the White-biased petition (BBBEE act: *M* = 5.17, *SD* = 1.18) was significantly above the scale midpoint (4), *t*(29) = 5.43, *p* < .001, *d* = 0.99. By contrast, support for the Black-biased petitions (Tertiary education: *M* = 1.87, *SD* = 1.01; Land reform: *M* = 1.77, *SD* = 1.01) was significantly below the scale midpoint, *t*(29) = -11.59, *p* < .001, *d* = -2.12 and *t*(29) = -12.16, *p* < .001, *d* = -2.22, respectively. Support for the arbitrary petitions (Water saving: *M* = 6.10, *SD* = 0.96; Drunk driving: *M* = 6.40, *SD* = 0.93) were significantly above the scale midpoint, *t*(29) = 11.99, *p* < .001, *d* = 2.19 and *t*(29) = 14.10, *p* < .001, *d* = 2.57, respectively. Given that participants’ responses were in line with the diagrams showing racial polarization for the petitions, it could be deduced that the manipulation afforded greater salience to racialized political identities.

### Minimal group empathic responses

Mixed factorial ANOVAs tested the influence of the between-group racial political salience manipulation (control vs manipulation), and the repeated-measures target team (Eagles vs Leopards) and target race (Black African vs White) variables on self-reported empathy (see S1 Table for extended ANOVA results). Empathy and counter-empathic responses are presented in Table 1.

**Table 1:** Self-reported empathy and counter-empathic responses.

			Empathic responses			Counter-empathic responses		
Racial political salience manipulation	Target team	Target race	Physical pain	Emotional distress	Positive events	Physical pain	Emotional distress	Positive events
Control group (*n* = 30)	Eagles	Black	6.86 (0.82)	7.41 (0.77)	7.10 (0.52)	2.28 (0.65)	2.04 (0.58)	1.66 (0.55)
		White	7.69 (0.76)	8.03 (0.72)	7.79 (0.75)	1.63 (0.49)	1.57 (0.54)	1.17 (0.28)
	Leopards	Black	6.33 (0.93)	6.97 (0.62)	6.78 (0.64)	2.96 (0.79)	2.39 (0.49)	2.12 (0.66)
		White	7.01 (0.65)	7.66 (0.56)	7.39 (0.72)	2.16 (0.53)	1.88 (0.48)	1.62 (0.57)
Manipulation group (*n* = 30)	Eagles	Black	7.03 (0.97)	7.45 (0.67)	7.13 (0.41)	2.14 (0.52)	2.04 (0.48)	1.70 (0.37)
		White	7.93 (0.95)	8.18 (0.73)	7.80 (0.81)	1.70 (0.51)	1.59 (0.53)	1.23 (0.27)
	Leopards	Black	6.38 (0.90)	6.93 (0.78)	6.54 (0.48)	3.05 (0.90)	2.38 (0.42)	2.20 (0.66)
		White	7.04 (1.00)	7.53 (0.72)	7.28 (0.50)	2.15 (0.49)	1.75 (0.45)	1.58 (0.53)

*Note*: Means are presented with standard deviations in parentheses. Empathic responses (“sorry” ratings in response to physically painful and emotionally distressing events, and “good” ratings in response to positive events) and counter-empathic responses (“good” ratings in response to physically painful and emotionally distressing events, and “sorry” ratings in response to positive events) ranged from 1 (*not at all*) to 9 (*very much*). Empathic and counter-empathic responses toward physically painful, emotionally distressing, and positive events differed significantly for target teams (Eagles vs. Leopards) and target race groups (Black vs. White). No significant differences were observed between the control and manipulation groups.

#### Self-reported empathic responses

**Fig 3:**
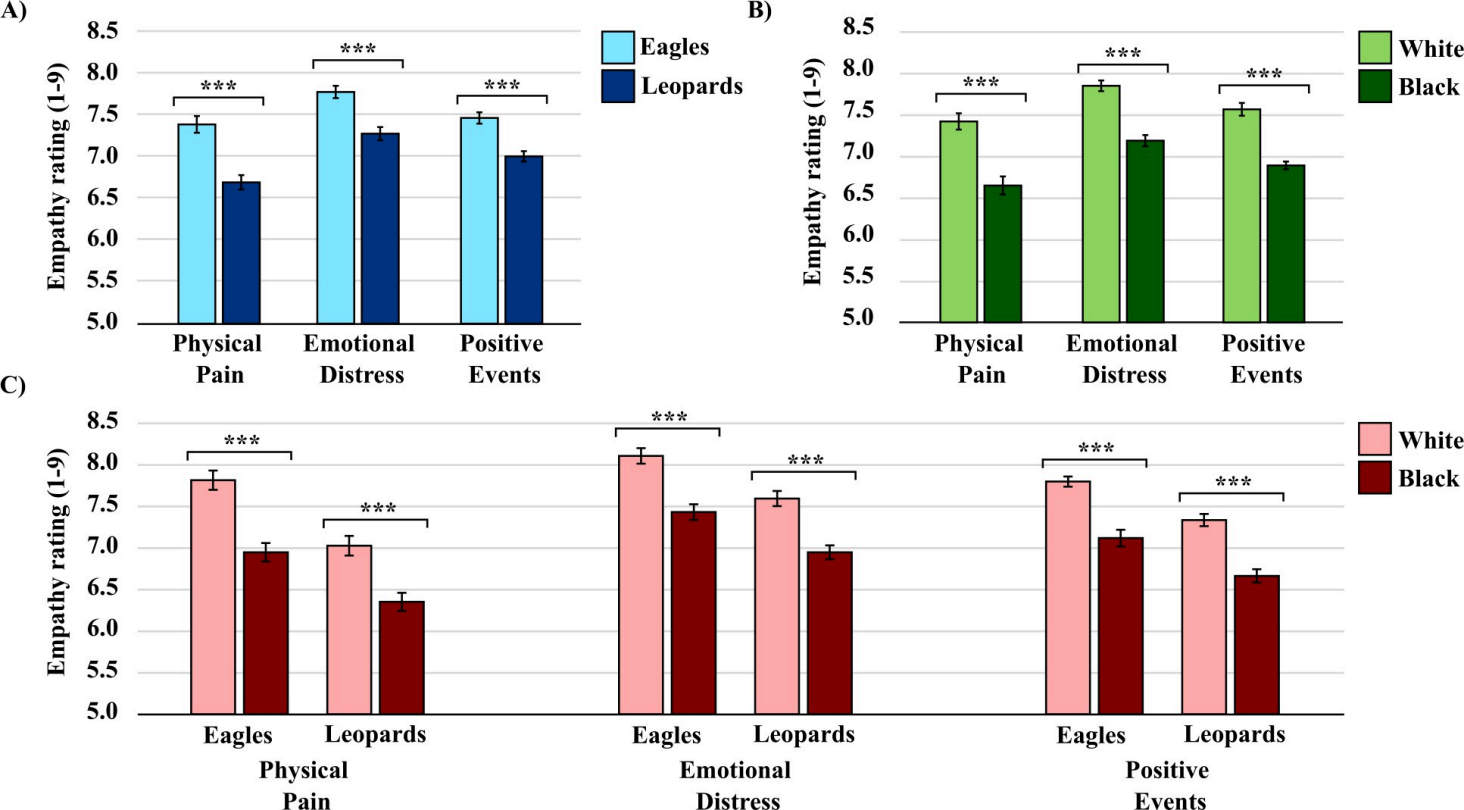
Self-reported empathy ratings in response to physically painful, emotionally distressing, and positive events. Empathy ratings for (A) ingroup (Eagles) versus outgroup (Leopards) teams, for (B) White versus Black African team members, and for (C) Team x Race interactions. Empathy ratings in response to physically painful and emotionally distressing events were determined by how “sorry” the events made participants feel, whereas empathy ratings in response to positive events were determined by how “good” the events made participants feel. Error bars indicate standard error of the mean. ***p < .001.

A 2 (racial political salience: control vs manipulation) x 2 (target team: Eagles vs Leopards) x 2 (target race: Black vs White) mixed-factorial ANOVA detected significant main effects for target team in response to physically painful, *F*(1, 58) = 89.97, *p* < .001, ƞ^2^ = .61, emotionally distressing, *F*(1, 58) = 20.25, *p* < .001, ƞ^2^ = .26, and positive events, *F*(1, 58) = 30.68, *p* < .001, ƞ^2^ = .35. As anticipated, participants felt more “sorry” for ingroup team members (Eagles) in response to physically painful and emotionally distressing events, and more “pleasure” when ingroup team members (Eagles) experienced positive events, than for outgroup team members (Leopards) (see Fig 3A).

We also detected significant main effects for target race in response to physically painful, *F*(1, 58) = 43.91, *p* < .001, ƞ^2^ = .43; emotionally distressing, *F*(1, 58) = 65.90, *p* < .001, ƞ^2^ = .53; and positive events, *F*(1, 58) = 73.64, *p* < .001, ƞ^2^ = .56. On average, participants felt more “sorry” for White individuals in response to physically painful and emotionally distressing events, and more “pleasure” when White individuals experienced positive events, than for Black African individuals (see Fig 3B). Contrary to our predictions, we did not observe significant interaction effects between target team and target race, indicating that the racial bias in empathic responding was similar in size in both ingroup and outgroup teams.

Finally, the mixed-factorial ANOVAs did not detect any significant main effects for the racial political salience manipulation in response to physically painful, *F*(1, 58) = 0.53, *p* = .469, ƞ^2^ = .01, emotionally distressing, *F*(1, 58) = 0.00, *p* = .952, ƞ^2^ = .00, or positive events, *F*(1, 58) = 0.63, *p* = .430, ƞ^2^ = .01. Contrary to our expectations, these results suggest that our manipulation did not exacerbate any racial empathy biases amongst participants. We did not observe any other significant interaction effects (see Fig 3C).

#### Self-reported counter-empathic responses

**Fig 4:**
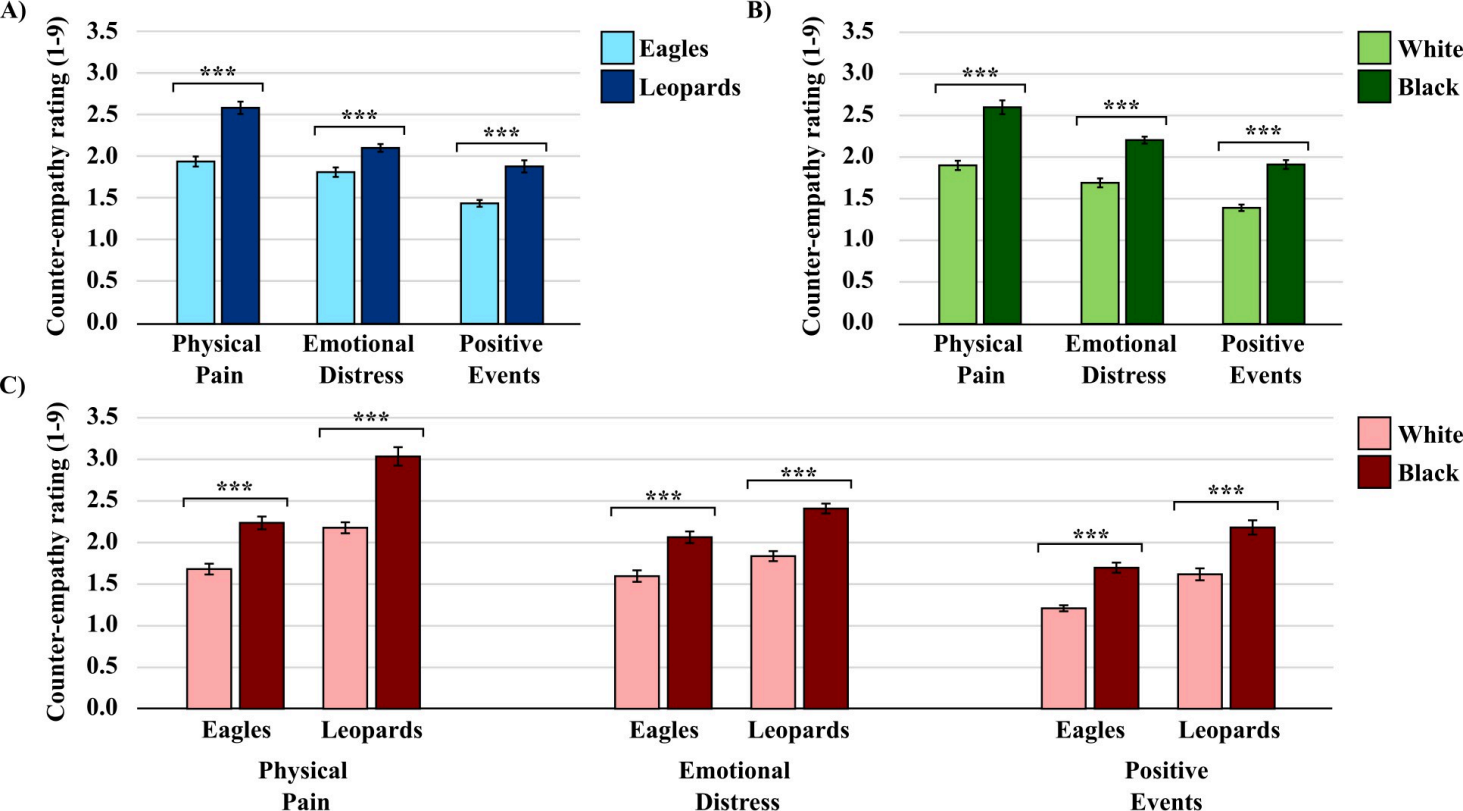
Self-reported counter-empathic ratings in response to physically painful, emotionally distressing, and positive events. Counter-empathic ratings for (A) ingroup (Eagles) versus outgroup (Leopards) teams, for (B) White versus Black African team members, and for (C) Team x Race interactions. Counter-empathic ratings in response to physically painful and emotionally distressing events were determined by how “good” the events made participants feel, whereas counter-empathic ratings in response to positive events were determined by how “sorry” the events made participants feel. Error bars indicate standard error of the mean. ***p < .001.

A 2 (racial political salience: control vs manipulation) x 2 (target team: Eagles vs Leopards) x 2 (target race: Black vs White) mixed-factorial ANOVA on counter-empathic responses detected a significant main effect for target team in response to physically painful, *F*(1, 58) = 103.09, *p* < .001, ƞ^2^ = .64, emotionally distressing, *F*(1, 58) = 16.08, *p* < .001, ƞ^2^ = .22, and positive events, *F*(1, 58) = 28.02, *p* < .001, ƞ^2^ = .33. On average, participants expressed more *Schadenfreude* in response to the physical pain and emotional distress of outgroup team members (Leopards), and more *Glückschmerz* when outgroup team members (Leopards) experienced positive events, compared to ingroup team members (Eagles) (see Fig 4A).

We again observed significant main effects for target race in response to physically painful, *F*(1, 58) = 81.74, *p* < .001, ƞ^2^ = .58, emotionally distressing, F(1, 58) = 68.56, *p* < .001, ƞ^2^ = .54, and positive events, *F*(1, 58) = 149.57, *p* < .001, ƞ^2^ = .72. On average, participants expressed more *Schadenfreude* in response to Black African individuals’ physical pain and emotional distress, and more *Glückschmerz* when Black African individuals experienced positive events, compared to White individuals (see Fig 4B).

We also detected a significant target team x target race interaction in response to physically painful events, *F*(1, 58) = 11.35, *p* = .001, ƞ^2^ = .16. In line with predictions, post-hoc comparisons indicated that participants’ “pleasure” ratings for Black African compared to White team members were significantly greater for the outgroup (Leopards) compared to the ingroup (Eagles) team (see Fig 4C).

Finally, we again did not detect a significant main effect for the racial political salience manipulation in response to physically painful, *F*(1, 58) = 0.00, *p* = .972, ƞ^2^ = .00, emotionally distressing, *F*(1, 58) = 0.18, *p* = .673, ƞ^2^ = .00, or positive events, *F*(1, 58) = .20, *p* = .653, ƞ^2^ = .00. These results suggest that ingroup race biases with regard to counter-empathic responses were not exacerbated by the manipulation.

### Individual differences

We examined the extent to which individual difference characteristics, including motivation to respond without prejudice (as measured by IMS/EMS scores) and perceived Black and White historical suffering, contributed to empathy in response to Black African target individuals. For these analyses we collapsed empathy ratings across the control and manipulation conditions (for a comparison of individual difference scores between the control and manipulation conditions, see S2 Table). Furthermore, given that we observed racial empathy biases similar in magnitude for both ingroup and outgroup teams, we collapsed empathic responses across the Eagles and Leopards teams for each event type. Descriptive statistics and variable intercorrelations are presented in Table 2 (separate correlations for each team are presented in S3A and S3B Table).

**Table 2:** Individual difference measures: Descriptive statistics and variable intercorrelations.

Measures	1.	2.	3.	4.	5.	6.	7.
1. IMS	-						
2. EMS	-.89***	-					
3. Black historical suffering	.56***	-.54***	-				
4. White historical suffering	-.47***	.47***	-.72***	-			
5. Empathy: Physical pain	.75***	-.70***	.52***	-.40**	-		
6. Empathy: Emotional distress	.52***	-.42***	.16	-.12	.33*	-	
7. Empathy: Positive events	.24	-.29*	.22	-.13	.34**	.15	-
*M*	6.63	6.49	7.30	1.78	6.65	7.19	6.89
*SD*	0.96	0.93	0.89	0.90	0.84	0.52	0.36

*Note*. Empathy ratings reflect scores for Black African target individuals collapsed across the Eagles and Leopards teams.

IMS = Internal Motivation Scale, EMS = External Motivation Scale

*p < .05

**p < .01

***p < .001.

As anticipated, higher IMS scores were associated with greater empathy in response to Black African targets in physical pain and emotional distress, whereas higher EMS scores were associated with less empathy in response to Black African targets in physical pain and emotional distress, and less pleasure when they experienced positive events. In turn, greater perceptions of Black historical suffering were positively associated with empathy for Black African targets in physical pain, whereas greater perceptions of White historical suffering were negatively associated with empathy for Black African targets in physical pain.

Next, we performed a series of simultaneous regressions with (i) IMS and EMS scores and (ii) perceptions of Black and White historical suffering as predictors of empathy in response to Black African targets experiencing physically painful, emotionally distressing, and positive events (see Table 3; for extended regression results, see S4 Table). Here IMS emerged as the only significant predictor of empathy for both physically painful and emotionally distressing events (βs > .58, *p*s < .01). Results of these analyses thus show that White participants’ internal motivation to respond without prejudice contributes significantly to greater empathy in response to the misfortunes experienced by Black African individuals.

**Table 3:** Individual difference measures: Simultaneous regressions predicting self-reported empathy for different event types.

	Physical Pain		Emotional Distress		Positive Events		Collinearity Statistics	
	*R*^*2*^ = .58, *p* < .001		*R*^*2*^ = .30, *p* = .001		*R*^*2*^ = .10, *p* = .217		Tolerance	VIF
	B	β	B	β	B	β		
IMS scores	.51	.58**	.41	.75**	-.06	-.15	0.20	5.13
EMS scores	-.10	-.12	.08	.14	-.15	-.38	0.20	5.02
Black historical suffering	.15	.16	-.08	-.14	.07	.18	0.42	2.39
White historical suffering	.03	.04	.04	.07	.04	.11	0.47	2.12

*Note*. Empathy ratings reflect scores for Black African target indivduals collapsed across the Eagles and Leopards teams.

IMS = Internal Motivation Scale; EMS = External Motivation Scale; VIF = variance inflation factor

*p < .05

It should be noted that due to the high correlations between IMS and EMS scores (-.89) and Black and White Historical suffering (-.72), the estimated regression parameters could be affected negatively by collinearity. We therefore examined collinearity statistics for these variables in our sample (see Table 3). Our analysis showed that collinearity for our measures of historical suffering were negligible, whereas the IMS and EMS variables showed moderate collinearity, with a tolerance of .20 for both and a variance inflation factor (VIF) of 5.13 and 5.02, respectively. Although these conditions are not ideal, they do fall within the acceptable guidelines of tolerance (> 0.1) and VIF values (< 10) [85, 86].

## Discussion

In the present study, we examined whether recategorization into arbitrarily defined groups would be effective in bridging empathy biases constructed under generations of socio-historical and asymmetric conflict. Specifically, we investigated whether the recategorization of White individuals into mixed-race (Black African and White) teams using a non-competitive MGP design would ameliorate racial empathy biases towards ingroup team members in the South African context. As expected, we observed a team effect, with participants reporting significantly greater empathy and reduced counter-empathic responses for ingroup team members experiencing physically painful, emotionally distressing, and positive events. However, recategorization through team assignment did not override racial empathy bias towards ingroup team members for any event type: We observed significant racial biases in empathy and counter-empathic responses towards both ingroup and outgroup team members, with an internal motivation to respond without prejudice predicting greatest empathy for Black African target individuals. Interestingly, a manipulation that highlighted racialized political ideological differences between racial groups did not further exacerbate racial empathy bias in our sample, suggesting that such perceptions were already salient. These results imply that the MGP may be ineffective in overriding racial empathy bias, even at an explicit/behavioral level, in contexts characterized by overt power asymmetry and historical social hierarchies.

Findings from MGP studies report that recategorization with an arbitrarily-defined group may be sufficient in overriding behavioral and even automatic racial biases [14–17], also when it comes to empathy [32–35]. Given the significantly greater empathy we observed for racial ingroup versus outgroup members, even in the ingroup team, our results do not support these findings. What makes our results especially noteworthy is that racial empathy bias was observed despite task demands that emphasized attention to targets’ emotional state and psychological experience. In previous studies, active perspective taking not only increased the reported saliency of pain experienced by others, but also attenuated explicit and implicit expressions of racial empathy bias [33, 87].

Previous neuroimaging work suggests that racial empathy bias may persist within a minimal group context during the early perceptual stages of neural processing, but dissipates at the later cognitive stages of neural processing and subsequent explicit outcomes [32, 34, 35]. A possible reason for this finding is that humans may automatically detect and encode race as a by‐product of an adaptation to identify fellow group members in early perceptual stages of neural response [88, 89]. However, this bottom-up bias in neural perception can be modulated by top-down, cognitive evaluation that takes into account more complex social motivations and contexts [12, 35, 90]. Indeed, in our study we examined responses at this latter, explicit, level of evaluation.

In trying to interpret our findings, one could argue that beliefs regarding how much life hardship/pain Black African people have previously endured may have impacted empathic responses. Deska and colleagues [69] and Trawalter and colleagues [68] found that White participants empathized less with Black individuals when they assumed a priori that Black people experience less pain than White people. By this account, racial empathy bias is rooted in the perceived lower social status of Black people, and the stereotypical beliefs of White participants that Black people “toughen up” (perhaps as a coping mechanism) due to the various hardships typically associated with lower status [68, 69]. Our results do not support this interpretation, however, and suggests the opposite: greater perceived historical suffering of Black African people was associated with *greater* empathy in response to Black African target individuals experiencing physical pain.

Alternatively, racial empathy bias observed in our study might be rooted in the salience of social identity. Social identity theory [91] holds that group categorization shifts one’s self-concept from the individual to the collective, which often manifests as intergroup bias [51, 92]. While minimal groups have the potential to generate stronger group biases than naturally occurring social groups (e.g., gender, race) [93], these biases tend to be context sensitive. Moreover, ingroup favoritism tends to increase within naturally-occurring groups if subjects show a deeper attachment to those groups [94]. In South Africa, the hierarchical race categories forged by the apartheid government is still considered a primary constituent of identity [1, 5, 95, 96]. Given this racialized and asymmetric socio-political context, it is reasonable to assume that the minimal groups created in our study were not meaningful enough to shift saliency away from White identity towards the new ingroup.

Another factor that may exacerbate racial empathy bias is status threat. Intergroup threat theory [43] suggests that a threat to one’s group’s social status could increase the salience of ingroup identity, resulting in higher levels of intergroup bias [36, 51, 52]. In the South African post-apartheid context, White people lost their political power to the Black majority, yet largely continue to hold economic power and privilege [95, 96]. Such a shift in power may destabilize White status and challenge the legitimacy of the existing status quo, thereby augmenting intergroup threat [39, 97]. Highlighting racialized differences in political ideologies as we did in our manipulation condition, especially those pertaining to economic prosperity (e.g., affirmative action legislation and land expropriation without compensation), was thus anticipated to exacerbate racial empathy bias, potentially through increased intergroup status threat.

Yet, Black African team members’ supposed support of policies promoting their interests at the expense of White people appeared to have little effect on White participants’ expressed racial empathy bias. All this while participants clearly supported the White-biased petition (toning down of the BBBEE Act) and opposed the two Black-biased petitions (expansion of free tertiary education and increased land expropriation). Given the polarizing race-based narratives that are often shared in sensationalistic media reports (e.g., [56, 98, 99]), we believe it is possible that the ideologies highlighted by our manipulation were assumed by participants already, thereby muting the impact thereof. Future research should consider whether adapting the racial political salience manipulation to include views of racial outgroups that align with the political and ideological identities of the ingroup might mitigate against racial bias.

Our results further showed significant associations between individual differences and racial empathy bias. Here, an internalized motivation to be free of prejudice (as measured by IMS scores) held the most sway in the expression of empathy towards Black African individuals’ physical and emotional misfortunes. Additionally, we observed significant associations between expressed racial empathy and an external motivation to respond without prejudice (as measured by EMS scores) and perceived Black and White historical suffering.

Group position theory suggests that intergroup conflict is rooted in perceptions of entitlement and status threat [100]. Accordingly, perceptions of entitlement are fostered by dominant group members to justify unfair advantages begotten through an unequal social system, and anyone challenging this entitlement is consequently perceived as a threat [66, 101]. A sense of entitlement may explain why greater perceptions of White historical suffering in our sample were associated with lower empathy towards Black African individuals in physical pain, whereas greater acknowledgement of Black historical suffering was associated with greater empathy for their pain. Relatedly, perceived disenfranchisement may explain why White participants who felt compelled to act in non‐prejudiced ways by external pressures (as measured by the EMS) were more likely to hold greater perceptions of White historical suffering.

Some limitations of the present research are worth observing. First, while the presented results provide a glimpse into the complexities of cross-racial empathic responding in the South African context, the data are not necessarily representative of the broader population. Participants in the current study were young, White, educated, university students. Considering that only 7% of the South African population between 25 and 34 years old has a tertiary education [102], university students are considered a super elite demographic that typically enjoys a more privileged lifestyle compared to most South Africans. Young and educated White South Africans have also been associated with more progressive racial perceptions and motivations [67], so that the racial empathy biases we observed might be more substantial in the general population.

It should further be noted that the current study focused on the views and responses of a self-identified sample of White individuals as they pertained to Black African individuals, without intending to represent race as a binary construct. During apartheid, the hierarchical power division embedded in the system was not a simple, two-tier matrix that only distinguished “Whites” from “non-Whites”, but a complex system involving four racial groups (White, Black African, Coloured, and Indian people) [1]. This hierarchical system effectively created division between the different racial groups that continue to find expression today [103]. To better understand the complex social dynamics within the South African context, future studies should examine minimal group empathic responding also amongst historically disadvantaged groups.

Finally, in line with the original MGP boundary conditions [10, 91], the current study adopted a non-competitive MGP to examine intergroup empathy bias. Recently some MGP studies have introduced a salient competitive condition to examine its impact on intergroup bias [30, 104]. Across these studies, greater competitive threat from outgroup teams seemed to enhance ingroup identity, thereby increasing intergroup bias in empathic responding [23, 51, 105, 106]. Competition between minimal teams might thus, ironically, reduce racial bias within one’s own team. Future MPG research should consider including a competitive condition to investigate whether it would be effective in reducing racial empathy bias in contexts emerging from long-standing and asymmetrical social conflict.

## Conclusion

Despite significant progress in racial integration, particularly in higher education, post-apartheid South Africa continues to suffer from racial division [1, 96]. The present results suggest that racial identity continues to play a pivotal role in empathic responding in South Africa: non-competitive mixed-race minimal groups were unsuccessful in overriding explicit ingroup racial empathy biases in a sample of White South Africans, regardless of the event type. Our study further highlights the impact of individual beliefs and attitudes, with an internal motivation to respond without prejudice being most strongly associated with cross-racial empathy. These findings underscore the complexities of intergroup dynamics in contexts of historical power asymmetry. It further calls for more empirical attention devoted to understanding the impact that the continued use of race-based labels, fraught with historical preconceptions, has in such contexts [107]. Given increasing racial diversity in social and educational contexts, it is of intense interest to devote resources into the development of strategies aimed at bridging empathy divides in present-day South Africa.

### Positionality statement

Mindful that our social identities can influence our research [108], the authors wish to provide the reader with potentially relevant information about our social positioning: both MD and MF identify as White and South African. We believe referring to racial groups is warranted to unmask the continued social implications of race in the South African context, in full recognition of the intersectional complexities and heterogeneities within these artificial categories.

## Supporting information

S1 Text Supplementary methods. (DOCX)

S1 Fig Sample stimulus for dot-estimation task. (TIF)

S1 Table Empathic and counter-empathic responses: Extended ANOVA results. (DOCX)

S2 Table Variations in individual difference scores for control and manipulation conditions. (DOCX)

S3 Table Individual difference measures: Descriptive statistics and variable intercorrelations. A) Ingroup team (Eagles) targets. B) Outgroup team (Leopards) targets.
(DOCX)

S4 Table Individual difference measures: Extended results of simultaneous regressions predicting self-reported empathy for different event types. (DOCX)
