# Correction: (Not) part of the team: Racial empathy bias in a South African minimal group study

**DOI:** 10.1371/journal.pone.0296581

**Published:** 2024-01-02

**Authors:** The PLOS One Staff

There are errors in the Funding section. The correct Funding statement is: This work was supported by a grant MF received from Stellenbosch University, Subcommittee A, and funding MD received from the National Institute for the Humanities and Social Sciences (NIHSS). The funders had no role in study design, data collection and analysis, decision to publish, or preparation of the manuscript. There was no additional external funding received for this study.

The publisher apologizes for the error.

## References

[1] Deist M, Fourie MM (2023) (Not) part of the team: Racial empathy bias in a South African minimal group study. PLoS ONE 18(4): e0283902. 10.1371/journal.pone.0283902 37023090 PMC10079011

