# Supplementary material for: (Not) part of the team: Racial empathy bias in a South African minimal group study
Source: PLoS One. 2023 Apr 6;18(4):e0283902. doi: 10.1371/journal.pone.0283902 (PMC10079011; doi:10.1371/journal.pone.0283902)
Supplement: S1 Text — (DOCX) [file pone.0283902.s001.docx]

# S1 Text: Supplementary Methods

# **(Not) part of the team: Racial empathy bias in a South African minimal group study**

## Sample Information

Participants were recruited via convenience sampling and data collection was completed over a 1-month period. Data collection therefore did not run throughout a whole semester as suggested by the cover story. Undergraduate students from Stellenbosch University were sent an advertisement of the study via their institutional email addresses. The study was also advertised on notice boards on campus. The first 60 students identifying as White, between 18 and 30 years old, and with normal or corrected-to-normal vision (because the study included visual tasks), who responded to the advertisements were accepted as

participants for the study.

## Full List of Statements for the Ideological Manipulation

Below are a number of petitions on recent social issues that have been distributed online. For each of the petitions below, indicate how much you support the petition.

**1 (Not at all) 7 (Very much)**

**White-biased petition:**

- Urge parliament to tone down the Broad-based Black Economic Empowerment (BBBEE) Act, so that qualified White people stand a better chance of employment.

**Black-biased petitions:**

- Urge Stellenbosch University to expand free tertiary education for Black students at the expense of White students.
- Urge parliament to make urgent provision for the expropriation without compensation of White-owned land.

**Arbitrary petitions:**

- Urge the students and staff of Stellenbosch University to be more conscious of water saving efforts on campus and residences.
- Urge the students of Stellenbosch University to limit their alcohol consumption when going out and driving.

*Note*. The goal of the racial political salience manipulation was to make existing racially divisive political ideologies more salient. Petitions supposedly supported by White team members in the manipulation were termed “White-biased petitions” and petitions supposedly supported by Black African team members were termed “Black-biased petitions”. We employ the shortened terms “Black-biased petition” and “White-biased petition” simply to distinguish between these divisive political ideologies—they are not necessarily representative of the real-world political beliefs and ideologies of Black African and White South Africans per se.

## Full List of Descriptions for the Empathy Task

**Physically painful events:**

Kelly had a stomach ache after her lunch.

Anele cut her foot on broken glass.

Chris walked into a glass door and hurt his head.

Lungelo tripped and grazed his knees and elbows.

Bongani’s hand got bitten by a dog.

James’s fingers got slammed in a car door.

Lerato had a painful injection at the doctor.

Rebecca cut her finger slicing an apple.

**Emotionally distressing events:**

Nicola and her best friend haven’t spoken in months.

Asanda’s friend forgot it was her birthday.

Matt could not find a date for his dance.

Sipho was not invited to his soccer team braai.

Andile’s girlfriend broke up with him.

Adrian felt left out of a team project in class.

Thandi hasn’t seen her mother in a year.

Sarah’s friend forgot about their dinner date.

**Positive events:**

Sophie caught 6 green lights in a row driving to uni.

Alice’s housemate surprised her with her favourite meal.

Nico found a R50 note on the floor.

Jack won a bet with a friend.

Thobeka was home without plans when she got invited to a fancy party.

Nomvula got indoors just before it started pouring rain.

Mpilo found a valuable possession he thought he had lost.

Siyanda scored a goal for his soccer team.

## Problem-solving Task

1. Without lifting your pencil from the paper, show how you could join all 4 dots with 2 straight lines

Solution:

1. Divide this figure into four equal sections so that they are the same size and shape

Solution:

1. How many months have twenty-eight days in them?

Solution: *All months have 28 days in them*

1. Rearrange the following patterns to make familiar words:

ritshyt

flymia
lendraca

Solution:

= *thirsty*
= *family*

= *calendar*
