# Supplementary figures and images for: (Not) part of the team: Racial empathy bias in a South African minimal group study
Source: PLoS One. 2023 Apr 6;18(4):e0283902. doi: 10.1371/journal.pone.0283902 (PMC10079011; doi:10.1371/journal.pone.0283902)

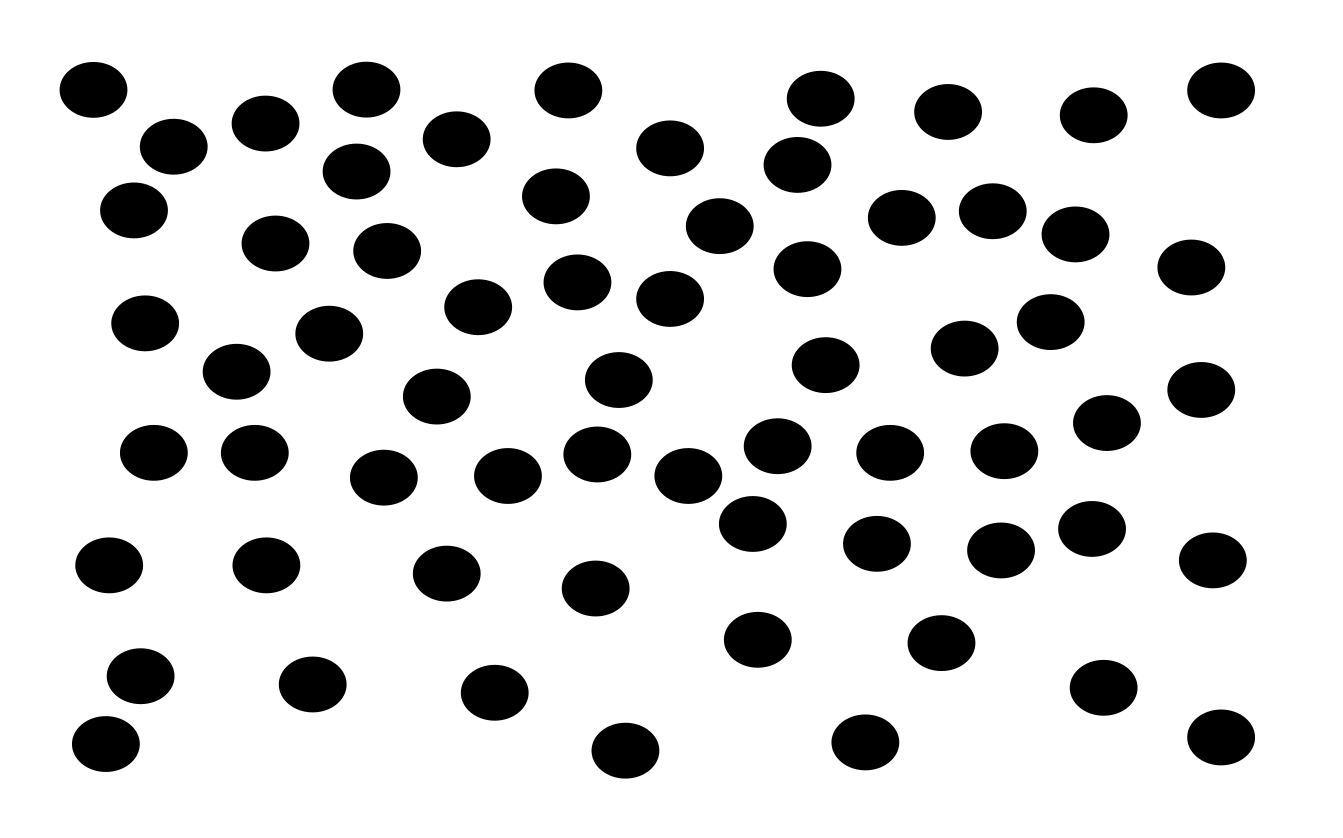

Supplement: S1 Fig — (TIF) [file pone.0283902.s002.tif]
