# Supplementary material for: (Not) part of the team: Racial empathy bias in a South African minimal group study
Source: PLoS One. 2023 Apr 6;18(4):e0283902. doi: 10.1371/journal.pone.0283902 (PMC10079011; doi:10.1371/journal.pone.0283902)
Supplement: S1 Table — (DOCX) [file pone.0283902.s003.docx]

**Table S1.** **Empathic and counter-empathic responses: Extended ANOVA results**

| **Variable/ interaction** | **Event** | **Empathic responses** | | | | | **Counter-empathic responses** | | | | |
| --- | --- | --- | --- | --- | --- | --- | --- | --- | --- | --- | --- |
|  |  | **df** | **Err** | **F** | ***p*** | **ƞ2** | **df** | **Err** | **F** | ***p*** | **ƞ2** |
| **Team** | Physical pain | 1 | 58 | 89.97 | **.000** | .61 | 1 | 58 | 103.09 | **.000** | .64 |
|  | Emotional distress | 1 | 58 | 20.25 | **.000** | .26 | 1 | 58 | 16.08 | **.000** | .22 |
|  | Positive events | 1 | 58 | 30.68 | **.000** | .35 | 1 | 58 | 28.02 | **.000** | .33 |
| **Race** | Physical pain | 1 | 58 | 43.91 | **.000** | .43 | 1 | 58 | 81.74 | **.000** | .58 |
|  | Emotional distress | 1 | 58 | 65.90 | **.000** | .53 | 1 | 58 | 68.56 | **.000** | .54 |
|  | Positive events | 1 | 58 | 73.64 | **.000** | .56 | 1 | 58 | 149.57 | **.000** | .72 |
| **Manipulation** | Physical pain | 1 | 58 | 0.53 | .469 | .01 | 1 | 58 | 0.00 | .972 | .00 |
|  | Emotional distress | 1 | 58 | 0.00 | .952 | .00 | 1 | 58 | 0.18 | .673 | .00 |
|  | Positive events | 1 | 58 | 0.63 | .430 | .01 | 1 | 58 | 0.20 | .653 | .00 |
| **Team * Manipulation** | Physical pain | 1 | 58 | 1.31 | .258 | .02 | 1 | 58 | 0.35 | .555 | .01 |
|  | Emotional distress | 1 | 58 | 0.72 | .400 | .01 | 1 | 58 | 0.37 | .547 | .01 |
|  | Positive events | 1 | 58 | 1.46 | .232 | .02 | 1 | 58 | 0.02 | .881 | .00 |
| **Race * Manipulation** | Physical pain | 1 | 58 | 0.01 | .915 | .00 | 1 | 58 | 0.14 | .708 | .00 |
|  | Emotional distress | 1 | 58 | 0.01 | .939 | .00 | 1 | 58 | 0.14 | .714 | .00 |
|  | Positive events | 1 | 58 | 0.10 | .752 | .00 | 1 | 58 | 0.34 | .559 | .01 |
| **Team * Race** | Physical pain | 1 | 58 | 2.71 | .105 | .04 | 1 | 58 | 11.35 | **.001** | .16 |
|  | Emotional distress | 1 | 58 | 0.07 | .793 | .00 | 1 | 58 | 1.37 | .246 | .02 |
|  | Positive events | 1 | 58 | 0.01 | .934 | .00 | 1 | 58 | 0.93 | .340 | .02 |
| **Team * Race * Manipulation** | Physical pain | 1 | 58 | 0.13 | .722 | .00 | 1 | 58 | 3.16 | .081 | .05 |
|  | Emotional distress | 1 | 58 | 0.89 | .350 | .02 | 1 | 58 | 0.64 | .429 | .01 |
|  | Positive events | 1 | 58 | 0.56 | .458 | .01 | 1 | 58 | 0.73 | .396 | .01 |
