# Supplementary material for: (Not) part of the team: Racial empathy bias in a South African minimal group study
Source: PLoS One. 2023 Apr 6;18(4):e0283902. doi: 10.1371/journal.pone.0283902 (PMC10079011; doi:10.1371/journal.pone.0283902)
Supplement: S2 Table — (DOCX) [file pone.0283902.s004.docx]

**Table S2. Variations in individual difference scores for control and manipulation conditions**

| **Variable** | **Racial political salience condition** | ***M*** | ***SD*** | **T-test results** | | |
| --- | --- | --- | --- | --- | --- | --- |
|  |  |  |  | *t* | *p* | *ƞ2* |
| IMS scores | Control | 6.47 | 0.98 | -1.28 | 0.21 | -0.33 |
|  | Manipulation | 6.79 | 0.91 |  |  |  |
| EMS scores | Control | 6.57 | 0.90 | 0.67 | 0.51 | 0.17 |
|  | Manipulation | 6.41 | 0.96 |  |  |  |
| Black historical suffering | Control | 7.27 | 0.91 | -0.29 | 0.77 | -0.07 |
|  | Manipulation | 7.33 | 0.88 |  |  |  |
| White historical suffering | Control | 1.73 | 0.87 | -0.43 | 0.67 | -0.11 |
|  | Manipulation | 1.83 | 0.95 |  |  |  |

*Note*. IMS= Internal Motivation Scale, EMS = External Motivation Scale

Motivation to respond without prejudice (as measured by IMS/EMS scores) and perceived Black and White historical suffering did not differ significantly between the control and manipulation conditions.
