# Supplementary material for: (Not) part of the team: Racial empathy bias in a South African minimal group study
Source: PLoS One. 2023 Apr 6;18(4):e0283902. doi: 10.1371/journal.pone.0283902 (PMC10079011; doi:10.1371/journal.pone.0283902)
Supplement: S3 Table — A) Ingroup team (Eagles) targets. B) Outgroup team (Leopards) targets. (DOCX) [file pone.0283902.s005.docx]

**Table S3. Individual difference measures: Descriptive statistics and variable intercorrelations**

1. **Ingroup team (Eagles) targets**

| **Measures** | **1.** | **2.** | **3.** | **4.** | **5.** | **6.** | **7.** |
| --- | --- | --- | --- | --- | --- | --- | --- |
| 1. IMS | - |  |  |  |  |  |  |
| 1. EMS | -.89^***^ | - |  |  |  |  |  |
| 1. Black historical suffering | .56^***^ | -.54^***^ | - |  |  |  |  |
| 1. White historical suffering | -.47^***^ | .47^***^ | -.72^***^ | - |  |  |  |
| 1. Empathy: Physical pain | .75^***^ | -.70^***^ | .53^***^ | -.43^***^ | - |  |  |
| 1. Empathy: Emotional distress | .54^***^ | -.43^***^ | -.05 | .05 | .38^**^ | - |  |
| 1. Empathy: Positive event | -.02 | -.02 | .01 | .09 | .15 | .07 | - |
| M | 6.63 | 6.49 | 7.30 | 1.78 | 6.95 | 7.43 | 7.12 |
| SD | 0.96 | 0.93 | 0.89 | 0.90 | 0.89 | 0.72 | 0.47 |

*Note*. Empathy ratings reflect scores for Black African target individuals in the Eagles team.

IMS= Internal Motivation Scale, EMS = External Motivation Scale.

***p* < .01. ****p* < .001.

1. **Outgroup team (Leopards) targets**

| **Measures** | **1.** | **2.** | **3.** | **4.** | **5.** | **6.** | **7.** |
| --- | --- | --- | --- | --- | --- | --- | --- |
| 1. IMS | - |  |  |  |  |  |  |
| 1. EMS | -.89^***^ | - |  |  |  |  |  |
| 1. Black historical suffering | .56^***^ | -.54^***^ | - |  |  |  |  |
| 1. White historical suffering | -.47^***^ | .47^***^ | -.72^***^ | - |  |  |  |
| 1. Empathy: Physical pain | .65^***^ | -.61^***^ | .43^***^ | -.31^*^ | - |  |  |
| 1. Empathy: Emotional distress | .22 | -.19 | .29^*^ | -.22 | .10 | - |  |
| 1. Empathy: Positive event | .31^*^ | -.35^**^ | .27^*^ | -.23 | .43^***^ | .25 | - |
| M | 6.63 | 6.49 | 7.30 | 1.78 | 6.35 | 6.95 | 6.66 |
| SD | 0.96 | 0.93 | 0.89 | 0.90 | 0.91 | 0.70 | 0.57 |

*Note*. Empathy ratings reflect scores for Black African target individuals in the Leopards team.

IMS= Internal Motivation Scale, EMS = External Motivation Scale.

**p* < .05. ***p* < .01. ****p* < .001
