# Supplementary material for: (Not) part of the team: Racial empathy bias in a South African minimal group study
Source: PLoS One. 2023 Apr 6;18(4):e0283902. doi: 10.1371/journal.pone.0283902 (PMC10079011; doi:10.1371/journal.pone.0283902)
Supplement: S4 Table — (DOCX) [file pone.0283902.s006.docx]

| **Physical Pain** | | | | | | |
| --- | --- | --- | --- | --- | --- | --- |
| *F*(4,55)=19.21*, R^2^* = .58, *p* < .001 | | | | | | |
|  | **B** | **Std. Error** | **β** | ***p*** | **95% CI for B** | |
|  |  |  |  |  | **Lower** | **Upper** |
| IMS scores | 0.51 | 0.17 | 0.58 | .005 | .16 | .85 |
| EMS scores | -0.10 | 0.18 | -0.12 | .554 | -.46 | .25 |
| Black historical suffering | 0.15 | 0.13 | 0.16 | .251 | -.11 | .40 |
| White historical suffering | 0.03 | 0.12 | 0.04 | .780 | -.20 | .27 |
| **Emotional Distress** | | | | | | |
| *F*(4,55)=5.88*, R^2^* = .30, *p* = .001 | | | | | | |
|  | **B** | **Std. Error** | **β** | ***p*** | **95% CI for B** | |
|  |  |  |  |  | **Lower** | **Upper** |
| IMS scores | 0.41 | 0.14 | 0.75 | .005 | .13 | .69 |
| EMS scores | 0.08 | 0.14 | 0.14 | .592 | -.21 | .36 |
| Black historical suffering | -0.08 | 0.10 | -0.14 | .428 | -.29 | .12 |
| White historical suffering | 0.04 | 0.09 | 0.07 | .671 | -.15 | .23 |
| **Positive Events** | | | | | | |
| *F*(4,55)=1.49*, R^2^* = .10, *p* = .217 | | | | | | |
|  | **B** | **Std. Error** | **β** | ***p*** | **95% CI for B** | |
|  |  |  |  |  | **Lower** | **Upper** |
| IMS scores | -0.06 | 0.11 | -0.15 | .614 | -.27 | .16 |
| EMS scores | -0.15 | 0.11 | -0.38 | .196 | -.37 | .08 |
| Black historical suffering | 0.07 | 0.08 | 0.18 | .380 | -.09 | .23 |
| White historical suffering | 0.04 | 0.07 | 0.11 | .563 | -.11 | .19 |

**Table S4.** **Individual difference measures: Extended results of simultaneous regressions predicting self-reported empathy for different event types**

*Note*. Empathy ratings reflect scores for Black African target individuals collapsed across the Eagles and Leopards teams.

IMS= Internal Motivation Scale, EMS = External Motivation Scale, CI = Confidence Interval
